# Properties of Rennet Gels from Retentate Produced by Cold Microfiltration of Heat-Treated and Microfiltered Skim Milk

**DOI:** 10.3390/foods13203296

**Published:** 2024-10-17

**Authors:** Jarosław Kowalik, Justyna Tarapata, Adriana Lobacz, Justyna Zulewska

**Affiliations:** Department of Dairy Science and Quality Management, Faculty of Food Science, University of Warmia and Mazury in Olsztyn, Oczapowskiego 7, 10-719 Olsztyn, Poland; justyna.tarapata@uwm.edu.pl (J.T.); adriana.lobacz@uwm.edu.pl (A.L.); justyna.zulewska@uwm.edu.pl (J.Z.)

**Keywords:** microfiltration, cold MF, β-casein, rennet coagulation, skim milk gel

## Abstract

This study investigated the production of rennet gels from β-casein-depleted retentates obtained through cold microfiltration (MF) of skim milk (SM) that was treated beforehand to ensure microbial safety. The treatments included thermization (65 °C, 20 s), pasteurization (72 °C, 15 s), and microfiltration (50 °C; 1.4 μm pore size). The reduction in β-casein content was 0.98, 0.51 and 0.90%, respectively. All treatments resulted in the partial aggregation of serum proteins, which were slightly concentrated in the retentates obtained post cold MF process. This aggregation, along with concentration effect, likely inhibited β-casein dissociation from casein micelles and permeation, particularly in pasteurized milk. Renneting and coagulation properties of the retentates were comparable to those of the respective SM samples, with no significant differences in syneresis, water-holding capacity, or protein hydration. Notably, the retentate from thermized SM, which showed the best performance with the highest β-casein reduction (0.98%), demonstrated shorter coagulation time compared to retentate from pasteurized milk or the corresponding unfiltered SM. Textural analysis revealed greater firmness, cohesiveness, and viscosity of retentate-based rennet gels compared to gels made from unfiltered SM, attributed to protein concentration during cold MF. Overall, this study successfully produced rennet gels from cold MF retentates without compromising their physicochemical properties.

## 1. Introduction

The composition of milk plays a critical role in determining its functional and technological properties. Recent advancements in membrane filtration, particularly cold microfiltration (MF), have enhanced the ability to selectively modify milk composition by the removal of constituents such as β-casein and serum proteins (SP), which are of interest in cheese making and other dairy applications [1]. In the dairy industry, the term ‘cold’ when applied to the MF process encompasses a wide range of temperatures, with temperatures below 20 °C (typically 4–20 °C) classified as low-temperature filtration [2]. Cold MF allows for the efficient separation of β-casein from casein micelles, as β-casein dissociates from micelles at temperatures below 10 °C. Upon cold storage, β-casein dissociates from micelles into the serum phase, accounting for about 55% of the total increase in serum casein after 24 h at 5 °C [3]. Various membrane filtration techniques have been developed to recover β-casein-enriched whey from micellar casein or skimmed milk, achieving recoveries between 2 and 20% of the total β-casein [4,5,6].

The growing interest in casein fraction purification, especially β-casein, has sparked advancements in cold MF technology. Improvements in the rehydration properties of micellar casein concentrate and optimizing the casein ratio in infant formula base ingredients are some of the key benefits of β-casein separation [6,7]. Fractionating β-casein could be beneficial for humanizing the protein profile of infant formula (IF), especially when paired with de-phosphorylation [8]. This has the potential to increase the value of the production chain and the finished products.

In cheese production, β-casein depletion can reduce bitterness, as peptides derived from β-casein are thought to contribute to this perception [9]. Moreover, the removal of β-casein significantly affects rennet coagulation, a crucial factor in cheese making. Some studies have reported weakened rennet-induced gels with reduced β-casein content [10]. Other have shown no significant impact on cheese composition or yield with β-casein removal (1.83–4.25% reduction) [1]. These discrepancies highlight the need for further research to fully understand the influence of cold MF on rennet coagulation and gel texture. Moreover, the cold MF of milk appears to be economically justified, as β-casein-rich permeate has the potential to enhance the quality of infant formula. Additionally, β-casein and SP-depleted retentate can improve cheese flavor, ultimately resulting in higher profits for producers while offering consumers improved premium products.

In parallel, SP removal before cheese making does not significantly alter cheese composition since most SP is lost in the whey [11,12]. However, SP-depleted milk can enhance cheese yield and quality, as observed in mozzarella production [13]. In contrast, the presence of denatured SP in heat-treated MF retentates may hinder curd formation by inhibiting chymosin activity [14,15]. Heat treatment, commonly used to ensure milk safety, alters the protein structure of milk, particularly affecting SP. Pasteurization (72 °C for 15 s) denatures a portion of SP, and this denaturation increases with higher temperatures and longer times [16]. Denatured SP forms complexes with κ-casein, impairing rennet coagulation and influencing cheese properties. The application of milder heat treatments, such as thermization or membrane sterilization of milk before cold MF, may improve the filtration efficiency and coagulation properties of retentate by reducing the thermal protein denaturation. Understanding how cold MF influences rennet coagulation properties of retentates from both heated and unheated milk remains crucial for optimizing dairy processing.

Previous work by Zulewska et al. [17] explored how different treatments of skim milk—thermization, pasteurization, and MF for bacteria removal—affected SP and β-casein removal, as well as the overall efficiency of cold MF. Building on this foundation, the present study aims to investigate the impact of these treatments on renneting and coagulation times of cold MF retentates, as well as the physicochemical and textural properties of retentate-based rennet gels.

## 2. Materials and Methods

### 2.1. Materials

Skim milk was obtained through centrifugation (45 °C; model LWG20 centrifuge, Spomasz, Gniezno, Poland) of raw bovine milk provided by the University of Warmia and Mazury (UWM in Olsztyn) Experimental Station in Bałdy (Poland).

### 2.2. Experimental Design and Cold Microfiltration

**Figure 1:**
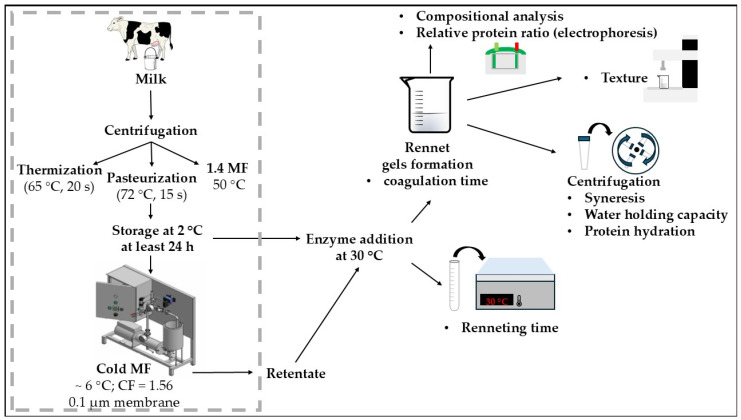
Experimental design: CF—concentration factor, MF—microfiltration, 1.4 MF—bacteria removal through microfiltration with 1.4 μm membrane (50 °C). The area outlined by the dashed line reflects the part of the study conducted as described in the study by Zulewska et al. [17].

The experimental design is illustrated in Figure 1. A skim milk batch of 60 kg was split into 3 sub batches, which were subjected to different heat treatment or bacterial MF (no heat treatment, control sample). First portion (20 kg) of skim milk was thermized at 65 °C for 20 s. Another batch (20 kg) was pasteurized at 72 °C for 15 s. Heat treatment was performed using a plate heat exchanger (model P20-HB; Alfa Laval, Lund, Sweden). The third portion (~4 kg) was microfiltered using 1.4 μm ceramic membrane (ET1-070, α-albumina; Pall Corp.) at 50 °C for bacteria removal (1.4 MF). The MF system was a benchtop crossflow pilot unit (Pall Membralox XLAB 5, Pall Corp., Port Washington, NY, USA). Treated milk was cooled and stored at 2 °C (for at least 24 h) before being subjected to cold MF process, allowing for the temperature-dependent dissociation of β-casein from casein micelles, which was expected to enhance fractionation efficiency during cold MF. Then, one batch of milk (1.2 L) was microfiltered at ~6 °C, using the same system as for bacterial removal, this time equipped with 0.1 μm nominal pore diameter ceramic membrane (Membralox; ET1-070, α-albumina, Pall Corp.), until volume concentration factor of 1.5 was achieved. As a result of cold MF of thermized, pasteurized and 1.4 MF skim milk, retentates with β-casein content reductions of 0.98, 0.51, and 0.90% were obtained. The SP that passed through the membrane accounted for 6.5% of the initial content in milk across all produced retentates. The processing of each tested sample was performed over 4 days. Heat treatments and bacteria removal through MF were carried out on the first day. Cold microfiltration was performed starting on the second day (one variant each day). The experiment was replicated four times. The processing of samples subjected to different heat treatment conditions was performed in rotation to ensure that the average storage time before cold MF was the same across the four replicates. This study builds up on a larger project investigating how different treatments of skim milk—thermization, pasteurization and MF—for bacteria removal, applied prior to cold MF, affect SP and β-casein removal, and overall filtration process efficiency. A detailed description of the experimental design of skim milk treatment and filtration processes can be found in the previously published study by Zulewska et al. [17].

Skim milk samples after heat treatment and retentate samples obtained after cold MF of thermized, pasteurized and 1.4 MF skim milk (control) were immediately subjected to analyses. Then, rennet gels were produced. Samples of 40 mL of skim milk that underwent different heat treatments (control) as well as retentates obtained after cold MF of skim milk subjected to different heat treatment were transferred to 100 mL plastic containers and brought to 30 °C by heating in a water bath (Lauda, Alpha RA24, Lauda-Königshofen, Germany). Once coagulation temperature was achieved, the rennet gels were formed by the addition of the 1 mL of a 1.5% coagulant water solution (Chy-max, Chr. Hansen, Hørsholm, Denmark) to a beaker containing samples of either milk or retentates. Next, the samples were thoroughly mixed and incubated for 30 min in an incubator (Binder, BF53, Tuttlingen, Germany) at 30 °C. The enzymatic coagulation time, syneresis, water-holding capacity, protein hydration and texture attributes of the resulting gels were determined. Analyses of nitrogen compounds were also performed using the reference methods, the relative proportion between caseins were determined by means of SDS-PAGE electrophoresis, and the changes in ionic, total, and soluble calcium content were measured.

### 2.3. Compositional Analysis

Total solids (TS), total nitrogen (TN), noncasein nitrogen (NCN), and nonprotein nitrogen (NPN) were measured in skim milk subjected to cold MF and obtained retentates. These analyses were performed using standard methods: forced-air oven drying for TS (AOAC International, 2007; method 990.20; 33.2.44), and the Kjeldahl method for TN (AOAC International, 2007; method 991.20; 33.2.11), NCN (AOAC International, 2007; method 998.05; 33.2.64) with modifications according to Wojciechowski and Barbano (2015), and NPN (AOAC International, 2007; method 991.21; 33.2.12) [18]. True protein (TP) was calculated by subtracting NPN from TN and multiplying by a conversion factor of 6.38. Casein content was determined by subtracting NCN from TN and multiplying by 6.38. SP was calculated by subtracting NPN from NCN and multiplying by 6.38. All samples were analyzed fresh.

### 2.4. Calcium Measurements

#### 2.4.1. Total and Soluble Calcium

The determination of total and soluble calcium content was performed using atomic absorption spectrophotometry (ISO 8070:2007, IDF 119:2007 [19]; ICE 3000 series AA SPECTROMETER, Thermo Scientific, Loughborough, UK). The samples intended for the determination of soluble calcium were heated to 37 °C and then centrifuged for 35 min at 37 °C at 20,000× *g* (Thermo Scientific™ Sorvall™ RC 6 Plus Centrifuge). After centrifugation, the separated sediment was removed by filtration. The subsequent preparation steps were the same for both soluble and total calcium.

Weighed samples (ranging from 1 to 5 g) were incubated in the oven set at 60 °C for 6–12 h to dry the material. Then, the sample was ashed at 490 °C for 24 h, and 5 mL of 1 M HCl was added, followed by another ashing at 490 °C for 24 h. Next, 5 mL of 1 M HCl was added again to dissolve the resulting ash. The solution was quantitatively transferred to a 100 mL volumetric flask and the volume was made up with demineralized water. To stabilize the reading from the calibration curve, lanthanum (III) chloride was added to the solution so that it contained 1% La^3+^. Additionally, calcium standard solutions were also prepared. The transmission (% T) was read, and calcium content (X) in mg per 100 g of the sample was calculated using the following formula:

X = (A × 100)/B(1)

where:

- A—reading from the standard curve (mg Ca in 100 cm^3^);
- B—sample weight (g).

The total and soluble calcium content were determined in samples of skim milk (raw, thermized, pasteurized and subjected to bacterial MF process), as well as in the retentates obtained after cold MF of skim milk subjected to different heat treatments and 1.4 MF.

#### 2.4.2. Ionic Calcium

Concentration of ionic calcium was determined using a Mettler Toledo ion meter (Seven Multi AG, 8603, Schwarzenbach, Germany) equipped with a perfectION™ calcium electrode (Mettler Toledo, Greifensee, Switzerland). The measurement of ionic calcium involved recording the potential generated at the electrode module upon contact with the sample. This potential depended on the level of free calcium in the tested solution. The sample (50 mL) was heated to approximately 20 °C, and 2 mL of a standardizing solution (NaCl) was then added to stabilize the ionic strength. Before performing the measurements, the device was calibrated using calibration standards with calcium ion concentrations of 1, 10, and 100 mg/L.

### 2.5. Relative Proportions between Proteins

The relative percentage of casein and major SP (β-lactoglobulin and α-lactalbumin) were determined in skim milk and retentates by means of SDS-PAGE electrophoresis. The fresh samples (0.1 mL) were diluted with 0.9 mL of sample buffer (10 mM Tris-HCl, 1.0% SDS, 20% glycerol, 0.02% bromophenol blue tracking dye, 50 mM dithiothreitol; pH 6.8), and were stored frozen in plastic vials (Eppendorf microtubes, 3810X; Merck, Darmstadt, Germany). Before the analysis, the samples were thawed at room temperature and heated at 100 °C for 3 min. The skim milk (9 μL) and retentates (7 μL) were loaded onto a 12% polyacrylamide gel (Bio-Rad Laboratories Inc., Hercules, CA, USA), with the differing volumes used to ensure that all wells contained the same protein content. Each sample was tested in 3 replicates. Running, staining, and destaining of gels were performed according to the procedure described by Verdi et al. [20]. The gels were scanned with a USB GS 800 Densitometer, and relative proportions between proteins were calculated using Quantity One 1-D Analysis Software Version 4.6.3 (Bio-Rad Laboratories Inc.).

### 2.6. Renneting and Coagulation Time

Renneting time was measured in samples of skim milk that underwent different heat treatments (control) as well as in the retentates obtained after cold MF of skim milk subjected to various heat treatments and 1.4 MF. Coagulant solution was added to the samples as described in Section 2.2 Next, 2 mL of renneted sample was transferred to a test tube (9 mL) and incubated at 30 °C in water bath. Time from coagulant addition until first para-casein flocs were noticed was measured as renneting time.

Coagulation time of 40 mL samples was measured from the moment the coagulant was added to the milk until a gel-like solid structure was observed.

### 2.7. Properties of Rennet Gels

#### 2.7.1. Texture

The texture attributes of 40 mL gel samples were determined using a texture analyzer TA.XT plus (Stable Micro System, Godalming, UK). The sample was located centrally beneath a 20 mm cylindrical flat probe. Gel firmness, consistency, cohesiveness, and the index of viscosity were investigated using a back extrusion test (speed of 1 mm/s; distance of 15 mm). Firmness was expressed as the maximum force needed for pressing the probe into the sample. Cohesiveness was described as the maximum force needed to overcome the resistance of the sample while probe was returning to its initial position. Consistency and index of viscosity corresponded to the area under the force versus time curve plotted while the probe was penetrating the sample and returning to its initial position, respectively. Gel cohesiveness and index of viscosity were described by negative values, which was related to the direction of the probe movement.

#### 2.7.2. Syneresis, Water-Holding Capacity and Protein Hydration

Rennet gels were transferred to 50 mL centrifuge tubes, weighed and centrifuged (Heraeus Megafuge 16R centrifuge; Thermo Fisher Scientific, Waltham, MA, USA) at 1100× *g* for 10 min at 10 °C (1st centrifugation). Then, expelled whey was carefully decanted and weighed. Syneresis was determined as previously described by Harwalkar and Kalab [21] as the mass percentage of whey expelled from the gel. The pellet obtained after first centrifugation was re-centrifuged at 13,500× *g* for 30 min at 10 °C (2nd centrifugation), drained, weighed, frozen (−23 °C for at least 24 h) and lyophilized using a freeze-dryer Alpha 1-2 LD plus (Christ, Osterode am Harz, Germany) under constant parameters: pressure 0.37 mbar; ice condenser temperature −55 °C; time 22 h. The water-holding capacity of the gel was calculated as the mass percentage of pellet (obtained after 2nd centrifugation) in the gel [22]. The protein hydration was expressed as the ratio of grams of water in the pellet obtained after 2nd centrifugation to grams of solids in this pellet [22].

### 2.8. Statistical Analysis

The experiment was replicated four times, and all analyses were conducted in four replicates unless otherwise stated. To determine significant differences between treatments, all data were analyzed using ANOVA followed by Tukey’s post hoc test. Statistical analysis was performed using Statistica software (version 13.1, 1984–2016, StatSoft, Inc., Tulsa, OK, USA).

## 3. Results and Discussion

### 3.1. Compositional Analysis

#### 3.1.1. Fractionation of Components

**Figure 2:**
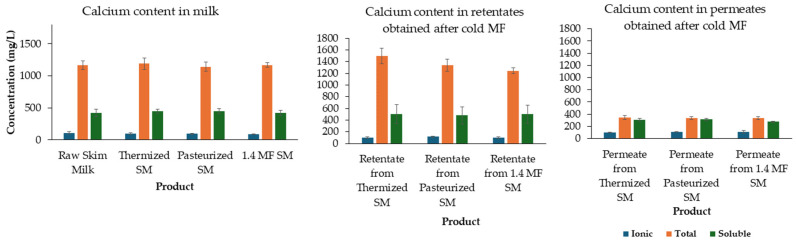
Calcium content in skim milk subjected to different heat treatments and the retentates and permeates obtained after cold microfiltration of treated skim milk; SM—skim milk; MF—microfiltration; 1.4 MF—bacteria removal through microfiltration with 1.4 μm membrane (no heat treatment).

The compositional analysis of retentates produced from skim milk subjected to either thermization, pasteurization or 1.4 MF, and the compositions of the respective feed materials (heat-treated and microfiltered skim milk) are summarized in Table 1. Crude protein content was similar (*p* > 0.05) in all tested retentates; however, the protein profile was different. The 1.4 MF process yielded skim milk with significantly lower (*p* < 0.05) casein content (2.53%) in comparison to 2.64 and 2.65% for pasteurized and thermized skim milk, respectively. Hence, the same relation was observed in the case of respective cold MF retentates, with retentate from 1.4 MF-treated milk exhibiting the lowest casein content of 3.36%, compared to approximately 3.64% in both pasteurized and thermized samples. The β-casein content was significantly lower in the retentate from thermized milk, representing approximately 35.00% of the total caseins, compared to 35.92% in retentate from pasteurized milk and 35.08% in 1.4 MF. Consequently, the lowest β-casein to α-casein ratio was also observed in the retentate from thermized skim milk, suggesting greater dissociation of β-casein from the micelle in thermized milk and its increased passage through the membrane during cold MF process. Additionally, SP constituted 0.64% (*w*/*w*) of the 1.4 MF milk, which was significantly higher (*p* < 0.05) than in milk subjected to thermization or pasteurization (0.61%). Again, a similar pattern was observed in resulting retentates with 1.4 MF retentate consisting of 0.90% SP, 0.84% for pasteurized and 0.86% for thermized skim milk. All produced retentates were characterized by similar total, ionic and soluble calcium content (Figure 2). The total calcium concentration was significantly higher in the retentates from thermized (1494 ± 130%) and pasteurized skim milk (1339 ± 105%) compared to their respective control samples (1190 ± 94 and 1144 ± 70%, respectively). Ionic and soluble calcium content were intact in this case, suggesting that only the concentration of insoluble form was increased. Cold MF did not affect the calcium concentration in the retentate from 1.4 MF skim milk.

During bacterial MF, the membrane effectively retains microorganisms while allowing milk to pass through. However, a significant limitation of this process is membrane fouling, where milk proteins accumulate on the membrane surface, forming a protein buildup. This results in a loss of protein mass, as some of the proteins become trapped on the membrane instead of passing through with the milk [23]. Tan et al. [24] reported that caseins contribute significantly to the fouling of membranes (1.4 μm pore diameter) during cold MF of skim milk, leading to irreversible binding to the membrane material. This binding is evident in the reduced casein content observed in this study in milk microfiltered at 50 °C using 1.4 μm membrane, where the caseins were major contributors to fouling.

Elevated levels of SP in retentates from thermized, pasteurized and 1.4 MF skim milk could be the result of protein aggregation. Heat treatment at temperatures above 60 °C leads to SP denaturation, coupled with the formation of SP polymer, either by itself or in combination with casein [25]. The degree of denaturation of individual protein fractions was reported at the following pasteurization temperatures: 75 °C for 5 min: α-casein (+0.5%), β-casein (+2.3%), κ-casein (−0.8%), β-lactoglobulin (−1.2%), α-lactalbumin (−6.2%); 95 °C for 5 min: α-casein (+8.1%), β-casein (+4.5%), κ-casein (−6.5%), β-lactoglobulin (−14.2%), α-lactalbumin (−14.9%) [25]. Denaturation causes the whey proteins to unfold, exposing reactive functional groups such as the free thiol groups in β-lactoglobulin. These groups can then react with other denatured whey proteins or caseins [26,27]. Although α-lactalbumin cannot undergo polymerization due to the absence of a free thiol group, its disulfide bonds still lead to denaturation through a thiol–disulfide bond exchange reaction [25]. Thus, thermal treatment affects the size distribution of proteins, leading to their retention in the retentate stream. The ratio of casein to true protein (CN%TP) was calculated as an indicator of the degree of denaturation of SP in this study.

Pasteurized and thermized skim milk was characterized by a similar CN%TP ratio, which was significantly higher (*p* < 0.05) compared to 1.4 MF milk, indicating the occurrence of denaturation in heat-treated samples. The CN%TP ratio increased by 1 and 1.1% for thermized and pasteurized skim milk, respectively, compared to raw skim milk. A similar ratio for 1.4 MF skim milk and raw skim milk (~80%) indicated that bacterial MF did not result in protein denaturation. Shear stress may also be a contributing factor to protein aggregation. Investigations of the minor whey protein bovine serum albumin (BSA) conducted by Chandavarkar [28] and Kim et al. [29] revealed that protein aggregates form under shear flow during cross-flow filtration. These aggregates subsequently deposit on the membrane surface. Furthermore, as suggested by Dzurec and Zall [30], heat treatment (74 °C, 10 s) caused β-casein to remain in the micelle rather than to solubilize and migrate into the milk serum as it does when unheated milk is cooled. One possibility is that whey protein molecules attach to the micelle’s surface as a result of heat-induced aggregation and physically block the casein molecules from leaving [30]. Based on these results, we can state that in the current study, denaturation and aggregation of protein in pasteurized milk inhibited β-casein dissociation during cold storage and consequently resulted in higher retention of β-casein in retentate fraction after a cold MF process. Moreover, MF does not remove micellar calcium, which mainly exists in the form of colloidal calcium phosphate (CCP) nanoclusters and a smaller proportion is also bound to phosphoserine and carboxyl residues of the caseins [31,32]. During the concentration of casein micelles, as the casein content increases, the calcium content also rises, since most of the calcium in milk is bound to micelles [33].

#### 3.1.2. The Efficiency of Protein Fractionation during Cold MF with Low Concentration Factor

In relation to process efficiency, understanding the composition and behavior of milk proteins is essential. According to the standard classification, milk proteins are divided into caseins (78–85% of the total protein mass), including α_s1_-casein (approximately 34%), α_s2_-casein (about 8%), β-casein (about 25%), κ-casein (about 9%), and γ-casein (around 4%); and whey proteins (15–25% of total milk proteins), including β-lactoglobulin (7–12% of milk proteins), α-lactalbumin (2–5%), immunoglobulins (1.3–2.7%), serum albumin (0.7–1.3%), and proteases and peptones (2–6%). The remaining composition of milk proteins includes proteins associated with fat globule membranes, accounting for about 0.1% [34]. In the study of Zulewska et al. [17], the cold microfiltration (0.1 µm, 6 °C) yielded the following retentates: thermized skim milk MF retentate with a 0.98% reduction in β-casein; pasteurized skim milk retentate with a 0.51% reduction in β-casein; and 1.4 MF skim milk retentate with a 0.90% reduction in β-casein. These were used in this research as a base for rennet gel production. Assuming that milk contains 2.5 g of casein per 100 g of milk, it can be estimated that β-casein (approximately 25%) constitutes 0.625 g/100 g of milk. Reductions of 0.98%, 0.51%, and 0.90% correspond to relatively small amounts (0.06125 g/1000 g, 0.031875 g/1000 g, and 0.05625 g/1000 g of milk, respectively). Although these amounts seem minor, considering that a dairy plant processes, for example, 10,000 kg of milk per hour, these values become significant, resulting in 612.5, 318.75, and 562.5 g of β-casein in the permeate, respectively. Moreover, the low-concentration MF process may be superior when compared to ultrafiltration, due to its potential for SP removal, when diafiltration is applied [35,36].

### 3.2. Rennet Coagulation and Moisture Retention in Gels

Renneting time ranged from 4.86 ± 0.90 s for the retentate obtained after cold MF of 1.4 MF skim milk to 6.07 ± 0.35 s for retentate from pasteurized skim milk (Table 2). Notably, only the retentate from pasteurized skim milk exhibited a significantly longer time for the formation of the first visible protein flocks (*p* > 0.05) compared to other retentates (from thermized and 1.4 MF skim milk). The concentration process resulted in a significantly delayed rennet action, as indicated by the extended renneting time for concentrates from thermized and pasteurized milk when compared to control heat-treated skim milk samples. Time to obtain a gel of desired firmness (coagulation time) was significantly (*p* < 0.05) shorter for retentate from thermized milk (20.00 ± 3.90 s) when compared to retentate from pasteurized milk (26.28 ± 2.06 s); however, this was only slightly reduced compared to 1.4 MF milk (22.00 ± 5.83 s). Despite significant differences in the onset of rennet gelation between concentrated and respective control samples, the coagulation time was not significantly (*p* > 0.05) altered after cold MF when compared to the control samples (Table 2). Furthermore, neither concentration nor heat treatment caused significant changes (*p* > 0.05) in syneresis, water-holding capacity, or protein hydration in the formed gels (Table 2).

It is well known that heat-induced SP denaturation can hinder the aggregation of para-casein by forming aggregates of β-lactoglobulin—paracasein, β-lactoglobulin—κ-casein and β-lactoglobulin—β-lactoglobulin [37]. Even though the SP content in pasteurized milk and retentate obtained from this milk was significantly lower than that in the 1.4 MF samples (Table 1), denatured SP may have partially impaired the rennet coagulation in the pasteurized samples. A similar mechanism could explain the differences in the time it takes for the first flocs to form between the control and concentrated samples, where the concentration of protein aggregates, formed as a result of denaturation, is higher after MF. Whereas retentates from thermized and pasteurized milk were characterized by similar SP content (Table 1), the coagulation time (set to cut time) was significantly lower for the thermized milk, in which β-casein reduction was significantly (*p* < 0.05) higher. Xia et al. [1] reported that rennet coagulation time (RCT) was significantly shorter for cheesemilk prepared from micellar casein concentrate powder with β-casein reduction of 1.83 or 4.25% when compared to cheesemilk from low-heat skim milk powder (RCT of 11.73, 11.37 versus 20.58 min, respectively). The reduction in β-casein content in MF retentates obtained in our study was much smaller (<1%) when compared to these of Xia et al. [1]. Moreover, the coagulation times showed in Table 2 were comparable with those noted for untreated samples in the cited study [1], explaining the limited impact of low-concentration-factor cold MF on rennet coagulation of retentate from heat-treated skim milk in the current study.

On the other hand, Holland et al. [36] suggested that the differences in gelation time are attributed to the differences in serum ions composition rather than to the presence or absence of β-casein or SP, though limited aggregation of the micelles still occurs. The concentrations of soluble and ionic calcium in skim milk and retentates produced in our study were comparable (Figure 2) and were not significantly altered by the cold MF process, which is why no major changes were observed in the time required for the gel to reach the cutting stage. It has been well established that calcium concentration plays a crucial role in the renneting process of milk. Gels with lower soluble calcium levels would be less firm and would take longer to form [38].

### 3.3. Texture of Rennet Gels

Textural attributes of rennet gels produced from either cold MF retentates or skim milk subjected to different heat treatment conditions are summarized in Table 3. Neither heat treatment nor the 1.4 MF process affected the texture of gels obtained either from retentates or respective skim milk samples. There was no significant difference (*p* > 0.05) in firmness, cohesiveness, consistency and index of viscosity values among retentates or among skim milk variants. Concentration had a significant impact (*p* < 0.05) on all texture parameters of the gels obtained from the retentates compared to the corresponding control samples. Only in the case of pasteurized milk there was no difference (*p* > 0.05) in the firmness of the curd obtained from the milk compared to the retentate.

There are relatively few studies on this topic, and the existing research shows some discrepancies. For instance, while it has been shown that β-casein-depleted milk forms softer gels with lower water-holding capacity than control skim milk gels [39], other findings suggest that lowering the β- to α-casein ratio in reconstituted milk protein concentrates can actually increase gel firmness, especially when protein concentration is adjusted to 2.4% caseins and calcium chloride is added [40]. The results summarized in Table 3 indicate that by reducing β-casein content by approx. 1%, the gels exhibited superior textural attributes (firmness, cohesiveness, consistency and viscosity). However, the increase in stiffness of the gel may also result from the concentration of proteins during the cold MF process (Table 1). The increase in protein content (to 6%) in cheese milk results in higher firming rate and narrower cutting window (time to cut the curd of optimum firmness) [41]. Given the low concentration factor achieved in our study, which raised the protein content to around 4.5%, the cold MF process likely maintained standard cheese making conditions. Therefore, no significant changes in monitoring the rennet coagulation process would be needed at this protein level, as in agreement with the findings of Panthi et al. [41].

## 4. Conclusions

This study explored the use of cold microfiltration (MF) to produce β-casein-depleted retentates from heat-treated (thermized, pasteurized and 1.4 MF—no heat treatment) skim milk for rennet gel formation. The reduction in β-casein content was relatively low (~1%). The research highlighted that heat treatment led to the partial denaturation of serum proteins and that these proteins were slightly concentrated in the retentates obtained after the cold MF process. This denaturation, combined with the concentration effects, likely contributed to the slight inhibition of β-casein dissociation from casein micelles, particularly in pasteurized milk. The renneting and coagulation properties of the retentates were similar to those of respective skim milk samples, with no significant changes observed in syneresis, water-holding capacity, or protein hydration. Only retentate obtained from thermized skim milk (characterized by the highest β-casein reduction of 0.98%) resulted in a shorter set-to-cut time when compared to pasteurized milk. Textural analysis of the rennet gels indicated enhanced firmness, cohesiveness, and viscosity when compared to gels made from skim milk, which can be attributed to the concentration of proteins during cold MF. Overall, rennet gels were successfully produced from retentates obtained through cold MF of heat-treated skim milk, without adversely affecting their physicochemical properties. Future studies could focus on further improvements in β-casein reduction and implications for different types of cheeses, and explore the scalability of the process. Moreover, some strategies to mitigate protein denaturation and aggregation could be undertaken to improve cold MF efficiency.

## Figures and Tables

**Table 1:** Composition (%, *w*/*w*) of skim milk subjected to different heat treatments and the retentates obtained after cold microfiltration of treated skim milk.

	TS	Fat	CP	NCN	NPN	TP	Cn	Cn%TP	Serum Proteins	*β*-Cn/*α*-Cn	*β*-Cn%Cn
Raw skim milk	9.35 ± 0.80	0.03 ± 0.02	3.49 ± 0.07	0.85 ± 0.03	0.20 ± 0.02	3.29 ± 0.06	2.64 ± 0.07	80.19 ± 0.93	0.65 ± 0.03	nd	nd
Skim milk:											
Thermized	9.14 ^a^ ± 0.22	nd	3.46 ^a^ ± 0.09	0.81 ^b^ ± 0.02	0.20 ^a^ ± 0.02	3.26 ^a^ ± 0.08	2.65 ^a^ ± 0.08	81.17 ^a^ ± 0.55	0.61 ^b^ ± 0.01	0.79 ^a^ ± 0.07	37.19 ^a^ ± 2.31
Pasteurized SM	9.22 ^a^ ± 0.70	nd	3.45 ^a^ ± 0.08	0.80 ^b^ ± 0.03	0.20 ^a^ ± 0.02	3.25 ^a^ ± 0.08	2.64 ^a^ ± 0.07	81.31 ^a^ ± 0.43	0.61 ^b^ ± 0.02	0.80 ^a^ ± 0.10	37.34 ^a^ ± 3.19
1.4 MF milk	9.02 ^a^ ± 0.09	nd	3.37 ^a^ ± 0.10	0.84 ^a^ ± 0.01	0.20 ^a^ ± 0.02	3.17 ^b^ ± 0.10	2.53 ^b^ ± 0.10	79.81 ^b^ ± 0.67	0.64 ^a^ ± 0.01	0.77 ^a^ ± 0.06	37.07 ^a^ ± 2.26
Retentate from:											
Thermized SM	10.21 ^a^ ± 0.52	nd	4.69 ^a^ ± 0.25	1.05 ^ab^ ± 0.04	0.19 ^a^ ± 0.03	4.49 ^a^ ± 0.23	3.64 ^a^ ± 0.23	80.86 ^a^ ± 0.97	0.86 ^b^ ± 0.03	0.71 ^b^ ± 0.04	35.00 ^b^ ± 1.45
Pasteurized SM	10.31 ^a^ ± 0.62	nd	4.67 ^a^ ± 0.27	1.03 ^b^ ± 0.04	0.19 ^a^ ± 0.02	4.49 ^a^ ± 0.25	3.64 ^a^ ± 0.23	81.15 ^a^ ± 0.86	0.84 ^b^ ± 0.03	0.75 ^a^ ± 0.03	35.92 ^a^ ± 1.18
1.4 MF SM	9.70 ^b^ ± 0.24	nd	4.45 ^a^ ± 0.03	1.10 ^a^ ± 0.04	0.20 ^a^ ± 0.02	4.26 ^b^ ± 0.03	3.36 ^b^ ± 0.06	78.89 ^b^ ± 0.90	0.90 ^a^ ± 0.03	0.72 ^a^ ± 0.04	35.08 ^a^ ± 1.86

Results are expressed as mean (*n* = 4) ± one standard devition; SM—skim milk, 1.4 MF—bacteria removal through microfiltration with 1.4 μm membrane (no heat treatment); TS = total solids; CP = crude protein (total N × 6.38); NCN = noncasein nitrogen × 6.38; NPN = nonprotein nitrogen × 6.38; TP = true protein (CP − NPN); casein = (CP − NCN); CN%TP = casein as percentage of true protein; serum proteins = (TP − casein), Cn—casein, nd—not determined; ^a,b^ Means in the same column (for either skim milk or retentates) not sharing a common superscript are different (*p* < 0.05).

**Table 2:** Renneting and coagulation time of skim milk subjected to different heat treatments and the retentates obtained after cold microfiltration of treated skim milk.

Product	Renneting Time (min)	Coagulation Time (min)	Syneresis (%)	Water Holding Capacity (%)	Protein Hydration (g Water/g TS)
Raw skim milk	3.14 ^a^ ± 0.63	17.85 ^a^ ± 2.67	56.98 ^a^ ± 2.25	12.79 ^a^ ± 1.12	2.31 ^a^ ± 0.61
Skim milk:					
Thermized	4.07 ^a,b,B^ ± 0.45	20.57 ^aA^ ± 4.47	55.49 ^aA^ ± 4.88	12.62 ^aA^ ± 1.42	2.49 ^aA^ ± 0.57
Pasteurized	4.85 ^aB^ ± 0.63	26.42 ^aA^ ± 7.93	54.69 ^aA^ ± 3.17	13.00 ^aA^ ± 1.87	2.54 ^aA^ ± 0.56
1.4 MF SM	4.00 ^bA^ ± 1.08	24.14 ^aA^ ± 1.07	59.47 ^aA^ ± 10.24	11.78 ^aA^ ± 0.59	2.47 ^aA^ ± 0.26
Retentate from:					
Thermized SM	5.00 ^bA^ ± 0.45	20.00 ^bA^ ± 3.90	52.54 ^aA^ ± 5.67	17.75 ^aA^ ± 3.59	2.32 ^aA^ ± 0.51
Pasteurized SM	6.07 ^aA^ ± 0.35	26.28 ^aA^ ± 2.06	50.86 ^aA^ ± 6.55	18.68 ^aA^ ± 2.99	2.30 ^aA^± 0.64
1.4 MF SM	4.86 ^bA^ ± 0.90	22.00 ^a,bA^ ± 5.83	51.93 ^aA^ ± 8.83	16.03 ^aA^ ± 3.64	2.29 ^aA^± 0.43

Results are expressed as mean (*n* = 4) ± one standard deviation, SM—skim milk, 1.4 MF—bacteria removal through microfiltration with 1.4 μm membrane (no heat treatment), ^a,b^ Means in the same column (for either skim milk or retentates) not sharing a common superscript are different (*p* < 0.05), ^A,B^ Means for skim milk versus retentate (for one heat treatment version) not sharing a common superscript are different (*p* < 0.05).

**Table 3:** Texture parameters of rennet gels produced from skim milk subjected to different thermal treatments and retentates obtained after cold microfiltration of treated milk.

Product	Firmness (N)	Consistency (Ns)	Cohesiveness (N)	Index of Viscosity (Ns)
Raw skim milk	1.56 ^a^ ± 0.18	14.68 ^a^ ± 0.97	0.35 ^a^ ± 0.08	0.37 ^aA^ ± 0.06
Skim milk:				
Thermized	1.78 ^aB^ ± 0.38	17.19 ^aB^ ± 3.99	0.37 ^aB^ ± 0.06	0.37 ^aA^ ± 0.05
Pasteurized	1.65 ^aA^ ± 0.31	15.86 ^aB^ ± 2.76	0.31 ^aB^ ± 0.07	0.34 ^aA^ ± 0.06
1.4 MF	1.59 ^aB^ ± 0.19	15.12 ^aB^ ± 2.81	0.33 ^aB^ ± 0.06	0.35 ^aA^ ± 0.04
Retentate from:				
Thermized SM	2.67 ^aA^ ± 0.70	26.22 ^aA^ ± 7.11	0.63 ^aA^ ± 0.08 ^a^	0.69 ^aB^ ± 0.16
Pasteurized SM	2.29 ^aA^ ± 0.62	23.08 ^aA^ ± 6.60	0.57 ^aA^ ± 0.11 ^a^	0.58 ^aB^ ± 0.07
1.4 MF SM	2.73 ^aA^ ± 0.89	22.38 ^aA^ ± 7.95	0.56 ^aA^ ± 0.13 ^a^	0.54 ^aB^ ± 0.07

Results are expressed as mean (*n* = 4) ± one standard deviation, SM—skim milk, 1.4 MF—bacteria removal through microfiltration with 1.4 μm membrane (no heat treatment), ^a^ Means in the same column (within either milks or retentates) not sharing a common superscript are different (*p* < 0.05), ^A,B^ Means for skim milk versus retentate (for one heat treatment version) not sharing a common superscript are different (*p* < 0.05).

## Data Availability

The original contributions presented in the study are included in the article, further inquiries can be directed to the corresponding author.
